# Grain supplementation of calves as an alternative beef production system to pasture-finished steers in Chilean Patagonia: meat quality and fatty acid composition

**DOI:** 10.1093/tas/txz188

**Published:** 2019-12-24

**Authors:** Francisco Sales, Leire Bravo-Lamas, Carolina E Realini, Raúl Lira, Noelia Aldai, Rodrigo Morales

**Affiliations:** 1 Instituto de Investigaciones Agropecuarias, INIA Kampenaike, Punta Arenas, Chile; 2 Grupo de Investigación Lactiker, Departamento de Farmacia y Ciencias de los Alimentos, Universidad del País Vasco (UPV/EHU), Vitoria-Gasteiz, Spain; 3 Meat Quality Team, AgResearch, Palmerston North, New Zealand; 4 Instituto de Investigaciones Agropecuarias, INIA Remehue, Osorno, Chile

**Keywords:** calf, grain, grass-fed, Patagonia, steer, supplementation

## Abstract

Beef production in Chilean Patagonia is based on steer finishing on pastures with low nutritive value. Grain supplementation for finishing calves on pasture arises as an attractive alternative to shorten the finishing phase and improve the production efficiency of the traditional system. The aim of this study was to compare meat quality and fatty acid (FA) composition of beef from steers and calves. Forty Angus cross steers were raised on pasture and slaughtered at 18 to 20 mo of age (448 ± 31.7 kg body weight). An additional group of 10 calves from similar genetics was weaned at 9 mo of age (303 ± 8.0 kg) and subsequently supplemented with 2.5 kg corn plus 1.0 kg commercial concentrate daily while on pasture during 47 d, until they reached slaughter weight (316 ± 13.9 kg). All animals were slaughtered on the same day and the Longissimus thoracis muscle was removed from each carcass for meat quality and FA profile analysis. Hot carcass weight from calves was lighter than steers (174 ± 7.9 vs. 224 ± 17.5 kg, *P* ≤ 0.001). Meat from calves was lighter (higher L*) and less red (lower a*). In addition, meat from calves showed lower level of yellow pigments in subcutaneous fat (lower b*) compared with meat from steers (*P* ≤ 0.001). Meat from calves was more tender than meat from steers (*P* ≤ 0.05), although both shear force values were low and within recommendations for high consumer acceptability. Meat from both production systems showed fat content and FA profiles within dietary recommendations for a healthy diet. However, meat from calves was leaner (1.10 ± 0.29 vs. 2.00 ± 0.72% fat, *P* ≤ 0.001). Meat from calves showed lower percentages of saturated (*P* ≤ 0.05) and monounsaturated (*P* ≤ 0.001) FAs and higher percentages of polyunsaturated and n-3 (*P* ≤ 0.001) FAs and conjugated linoleic acid (total and 9*c*,11*t*-18:2 isomer, *P* ≤ 0.001) than meat from steers. Grain supplementation of calves on pasture can reduce the finishing period by 8 to 10 mo at the expense of lighter carcasses with similar or improved meat quality characteristics compared with the traditional finishing of steers on pasture. Implementation of the alternative production system will depend mainly on feed costs and target carcass weights for specific markets.

## INTRODUCTION

Beef production in the Chilean Patagonia is traditionally based on extensive pasture systems. The harsh environment of this region during certain times of the year results in long grazing periods, and frequently, in leaner carcasses with poorer conformation and muscle and fat color compared with more intensive finishing systems (Keane and Allen, 1998). Thus, live animals are commonly transported from Patagonia to the more productive central-southern zone of Chile for breeding, fattening, and/or slaughter. In this context, there are concerns among farmers from different Patagonian regions about complying with new transport regulations contemplating high animal welfare standards. Therefore, alternative animal finishing systems to the traditional grazing in Patagonia, could contribute to the development of the meat industry in the region. Consistent production of high quality meat for the domestic and specially the international market, taking advantage of the increasing meat export potential of the country, is key for the future sustainability of the industry in the region (Abella et al., 2010).

Grain supplementation for finishing young steer-calves arises as an attractive alternative in order to improve the efficiency of the traditional Patagonian beef production system where steers are finished exclusively on pasture. However, grain inclusion in the animal diet may lead to changes in the characteristics of beef, potentially affecting some desirable quality attributes derived from pasture finishing such as the fatty acid (FA) composition of meat. Thus, the aim of this study was to compare meat quality characteristics and FA profile of beef from grass-fed steers (standard production system) vs. calves (alternative system) in Chilean Patagonia.

## MATERIALS AND METHODS

### Ethics

The study and all animal handling procedures were approved by the Instituto de Investigaciones Agropecuarias (INIA) Animal Ethics Committee according to the Animal Welfare Act 1999.

### Trial Design

The experiment was performed at INIA Kampenaike (52°42′S; 70°56′W; altitude 6 m.a.s.l.; annual rainfall 440 mm). The first group of animals had 40 Angus cross steers selected at weaning (6 to 7 mo of age; 247 ± 19.6 kg body weight (BW)), raised on a typical pasture from southern Patagonia, this is a natural pasture (*Festuca gracillima*–*Chiliotrichium diffusum*; Table 1) during winter time and meadow areas during the rest of the year (Table 1) and were slaughtered at 18 to 20 mo of age (448 ± 31.7 kg BW).

**Table 1.** Average chemical compositions of the diets (*n* = 3 by feed)

	Pasture of spring-summer	Pasture of winter	Corn	Concentrate
Dry mater (%)	52.3	43.5	71.5	89.0
Crude protein (%)	9.3	4.7	5.3	17.3
Metabolizable energy, Mcal/kg	2.2	1.8	3.5	2.4
Ash (%)	8.4	8.3	1.2	5.5
Ether extract (%)	2.6	1.9	2.5	5.6
Neutral detergent fiber (%)	55.6	66.9	6.4	39.0
Fatty acids (%)				
16:0	19.7	24.5	13.3	13.5
18:0	4.2	4.4	1.6	1.6
18:2n-6	22.6	15.7	53.9	43.2
18:3n-3	18.0	28.3	1.8	3.8

The second group corresponded to 10 noncastrated calves, from the same maternal herd as the first group. Calves were weaned at 7 mo of age (303 ± 8.0 kg BW) and received increasing levels of grain supplementation during 2 wk until reaching 2.5 kg corn, which was offered on a daily basis. In addition, from day 1, animals received 1.0 kg commercial concentrate (Cosetán, Iansa, Los Angeles, Chile) offered on a daily basis, as this product has minimal effect on ruminal pH. Supplementation lasted for 47 d, while animals were on winter pasture (Table 1), corresponding to the required time to reach the target slaughter weight (316 ± 13.9 kg BW), considering the average slaughter weight for young animals in Argentina, capable to reach an adequate subcutaneous fat thickness (Lucero-Borja et al., 2014). The composition of the commercial concentrate was: (g/kg, as-fed basis): Dehydrated sugar beet pulp (500), Beet molasses (250), Canola bran (140), wheat bran (98) and Urea (12). The chemical and FA compositions of the feed used in the experiment are shown in Table 1.

### Slaughter and Sampling Procedure

Steers and calves were slaughtered on the same day, following standard procedures at a commercial meat plant licensed for export. Briefly, animals were stunned with a captive bolt gun, followed by exsanguination an electrical stimulation was applied for 30 s. Afterwards, carcass was suspended from the Achilles tendon and eviscerated. Subsequently, the carcasses were dressed and chilled and entered the chillers (0°C) after the killing. Live and hot carcass weights were recorded at the meat plant. After 24 h postmortem chilling, cold carcass weight was recorded.

### Instrumental Color Analysis

The color was measured directly in the cold carcass. Instrumental color was measured at 3 random locations in the loin eye area of Longissimus thoracis (LT) muscle at 11th rib height. The average from measurements was recorded. Additionally, the external subcutaneous fat color of each carcass and same anatomical place were measured. Color measurements were recorded for L* (lightness: 0 = black to 100 = white), a* (redness/greenness: positive values, red; negative values, green), and b* (yellowness/blueness: positive values, yellow; negative values, blue) using a Minolta chromameter (CR-400; Minolta Inc., Osaka, Japan) with illuminant D_65_ and a 2° viewing angle. After wards, a section of the LT muscle from left side from the carcass between the 11th and 13th ribs was removed and vacuum packaged, aged for 21 d at 4 ± 2°C, and finally stored at −18 ± 2°C until analysis at the INIA Remehue Laboratory in Osorno, Chile. Thereafter, the samples were thawed and sliced with a knife in 4 steaks of 2.54 cm thick. The 2 cranial steaks were used for instrumental texture analysis and the 2 caudal steaks were used for chemical composition and FA analyses, respectively.

### Proximate Analysis

All the feed samples were transported refrigerated to the laboratory and were dried for 48 h at 60°C for chemical analyses. The chemical content of the feed samples was analyzed at the INIA Remehue Animal Nutrition and Environment Laboratory in Osorno, Chile. The dry matter, crude protein, ether extract, and ash were measured with the methods described by the AOAC (2005). The metabolizable energy and the neutral detergent fiber were determined according to Sadzawka et al. (2007).

Regarding meat samples, external fat was excised from meat samples with a knife before chemical analysis. For the proximate analysis approximately 100 g of meat samples without external fat were used. Moisture content was determined by drying at 105 ± 2°C until reaching constant weight (AOAC, 1990). Protein and ash were determined according to AOAC (2005) procedures and intramuscular fat content by Soxhlet extraction (AOAC, 2005).

### Instrumental Tenderness Analysis

Steaks used for texture analysis were covered with aluminum foil and cooked in a preheated oven (170°C; EKA, KF 620; Famava, Santiago, Chile) to an internal muscle temperature of 71°C, which was controlled by individual thermocouples (Sper Scientific LTD model 800024; Scottsdale, AZ, USA) inserted into the geometric center of each steak. After cooking, the steaks were wrapped with aluminum film and stored for 24 h at 4 ± 2°C. Subsequently, at least 6 cores (13 mm of diameter) were obtained from each steak for shear force analysis using a Warner–Bratzler shear blade with a triangular slot cutting edge to record the maximum Warner–Bratzler shear force in N. Crosshead speed was set at 200 mm/min whereas force load was 50 kg.

### Total FA Composition Analysis

Lipids were extracted from 1 g of freeze-dried and homogenized LT muscle using a mixture of chloroform–methanol (1:1, v/v; Folch et al., 1957). Details of this procedure have been published elsewhere (Aldai et al., 2012). Lipid aliquots (~10 mg) from each steak were methylated separately using acidic (methanolic HCl) and base (sodium methoxide) catalysis to ensure complete methylation of all lipids and avoid isomerization of conjugated linoleic acid (CLA), respectively. For quantitative purposes, 1 mL of internal standard (1 mg/mL of 23:0 methyl ester, n-23-M from Nu-Chek Prep Inc., Elysian, MN, USA) was added prior to methylation. The contents of FA methyl esters (FAME) were finally expressed as percentage (%) of total FAME.

The FAME were analyzed using a gas chromatograph (GC), equipped with a flame ionization detector (GC-2010 Plus; Shimadzu, Kyoto, Japan). A 100 m SP-2560 column (Supelco, Bellefonte, PA, USA) was operated at 2 complementary GC temperature programs that plateaued at 175 and 150°C (Kramer et al., 2008). In addition, a 100 m SLB-IL111 ionic liquid column (Supelco, Bellefonte, PA, USA) was used to confirm the identification of several biohydrogenation intermediates such as CLA isomers (Delmonte et al., 2011). With both columns, hydrogen was used as carrier gas with a constant flow rate of 1 mL/min, and the injector and detector temperature was set at 250°C. As previously detailed in Bravo-Lamas et al. (2016), for peak identification purposes, reference standards and retention times and elution orders reported in the literature were used.

### Statistical Analyses

The physicochemical data were analyzed using a General Linear Model procedure, to account for the unbalanced model, using the PROC GLM of the SAS statistical software package (SAS 9.4, Institute Inc., Cary, NC, USA), with steer and calves as experimental unit. The treatment (steers vs. calves) was included as fixed effect in the model. Least-square means were separated with Tukey’s studentized range test. The significance level was established at *P* ≤ 0.05.

## RESULTS

### Carcass Traits

Results of carcass traits are presented in Table 2. Young calves were lighter at slaughter (*P* ≤ 0.05) compared to steers resulting also in a lighter hot and cold carcass weight (*P* ≤ 0.05). However, carcass yield was significantly higher in calves compared to steers (*P* ≤ 0.001).

**Table 2.** Carcass and meat quality characteristics of the LT muscle from steers and calves

	Steers		Calves		
	Mean	SD	Mean	SD	*P*-value
Carcass parameters					
Live weight (kg)	417	30	310	13	≤0.001
Hot carcass weight (kg)	224	17	174	7	≤0.001
Hot yield (%)	53.6	1.7	56.0	1.4	≤0.001
Cold carcass weight (kg)	221	17	172	7	≤0.001
Cold yield (%)	53.1	1.7	55.4	1.4	≤0.001
Meat chemical composition (%)					
Moisture	75.4	1.1	76.6	0.8	0.002
Protein	20.6	0.7	20.4	0.7	0.077
Ash	1.1	0.15	1.2	0.3	0.323
Intramuscular fat	2.0	0.72	1.1	0.3	≤0.001
Meat quality parameters					
Shear force (kg)	2.22	0.25	1.97	0.30	0.009
Meat color					
L*	39.6	1.8	42.4	1.6	≤0.001
a*	25.5	1.8	23.2	1.4	≤0.001
b*	12.7	1.2	12.7	0.7	0.966
Fat color					
L*	67.0	1.9	65.9	1.5	0.086
a*	13.2	2.0	12.9	1.5	0.696
b*	17.2	1.5	14.1	0.8	≤0.001

### Chemical Composition and Instrumental Analyses

Chemical composition and instrumental analyses of meat are shown in Table 2. Moisture content of calf meat was higher than steer meat samples (*P* = 0.02), whereas similar levels of ash and protein were observed among samples (*P* > 0.05). The intramuscular fat content was significantly lower in steak samples coming from calves compared to steers (*P* ≤ 0.05). Shear force was lower (*P* ≤ 0.05) in calf meat compared to meat from steers. Regarding color attributes, meat from calves was less bright with lower L* value and less red with lower a* value than that of steers (*P* ≤ 0.05). Backfat surface from steers was more yellow showing a higher b* value than subcutaneous fat from calves (*P* ≤ 0.001).

### FA Composition

Total FAME were higher in the steer than in the calf meat samples (Table 3, *P* ≤ 0.001). Consequently, all the main groups of FA such as saturated FA (SFA), monounsaturated FA (MUFA) in both *cis*- and *trans*-configuration, n-3 and n-6 polyunsaturated FA (PUFA), and CLA isomers were higher (mg/100 g of fresh meat) in the LT muscle from steers than calves (Table 2, *P* ≤ 0.01). A similar 10*t*-/11*t*-18:1 ratio was obtained in meat from steers compared with calves.

**Table 3.** Total fatty acid content and summary of fatty acid composition (mg/100 g fresh meat) of the LT muscle from steers and calves

	Steers		Calves		
Fatty acid	Mean	SD	Mean	SD	*P*-value
Total FAME	1,940	764	897	281	≤0.001
∑SFA	903	393	402	143	≤0.001
∑BCFA	37.0	14.4	18.5	6.7	≤0.001
∑MUFA	770	328	312	108	≤0.001
∑cis-MUFA	719	308	292	101	≤0.001
∑trans-MUFA	50.9	20.5	19.8	6.5	≤0.001
10*t*-18:1	2.60	1.25	1.00	0.35	≤0.001
11*t*-18:1	23.3	9.2	9.05	3.19	≤0.001
10*t*-/11*t*-	0.110	0.021	0.112	0.018	0.853
∑PUFA	135	24	101	17	≤0.001
∑n~-6	85.3	15.1	66.5	10.7	≤0.001
18:2n-6	58.9	11.3	44.9	7.0	≤0.001
∑n~-3	32.8	6.3	22.8	4.6	≤0.001
18:3n-3	20.2	4.7	12.2	2.7	≤0.001
n-6/n-3	1.75	0.16	1.96	0.19	0.001
P/S	0.170	0.061	0.276	0.095	≤0.001
∑CLA	8.58	3.43	4.89	1.65	≤0.001
9*c*,11*t*-18:2	4.83	2.27	3.26	1.26	0.007

SD, standard deviation; SFA, saturated fatty acids; BCFA, branched-chain fatty acids; MUFA, monounsaturated fatty acids; *c*, *cis*; *t*, *trans*; PUFA, polyunsaturated fatty acids; P/S, PUFA/SFA

**Figure 1.**
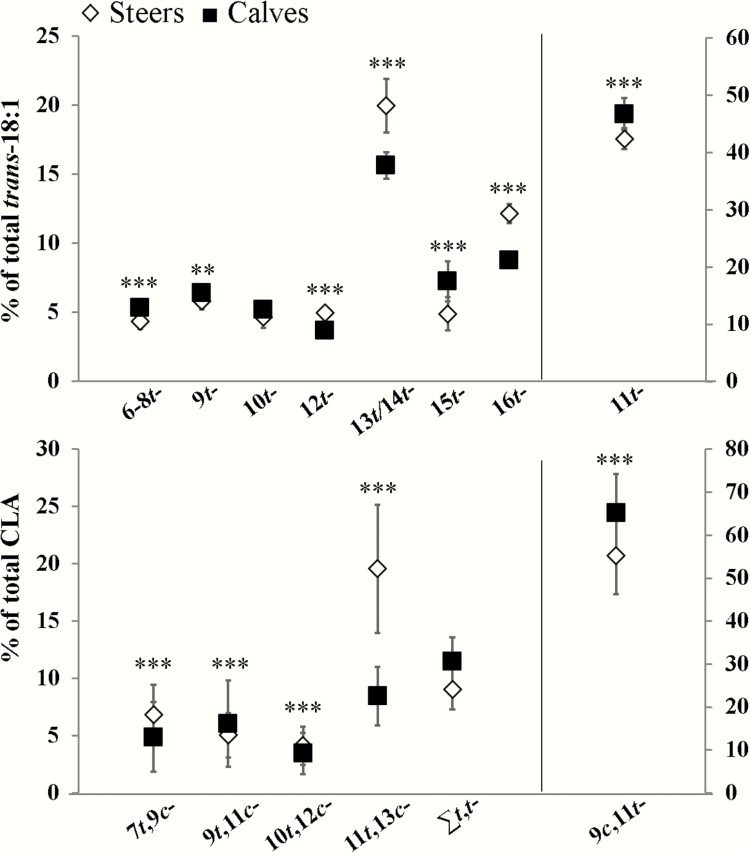
Relative content of individual (a) *trans*-18:1 and (b) CLA isomers of meat from steers and calves. Right axis shows vaccenic (11*t*-18:1) and rumenic (9*c*,11*t*-18:2) acid contents and left axis shows the rest of the isomers. Significant interactions is indicated with asterisks. **P ≤ 0.01; ***P ≤ 0.001.

A detailed FA profile, in percentage (%) basis, of muscle fat from steers and calves is presented in Tables 4 and 5 and Figure 1. Meat from extensively grass-fed steers had higher percentages of total SFA (45.8%) compared to meat from calves (43.9%, *P* ≤ 0.05, Table 4). For individual SFA, percentages of 14:0 and 15:0 were higher (*P* ≤ 0.05) in calf meat, whereas 18:0 and 20:0 were higher in steers’ muscle. No significant differences were found in total branched-chain FA (BCFA) percentages between both groups of samples, however, calf meat showed higher percentages of *iso*-17:0 and *iso*-18:0 and lower of *iso*-15:0 (*P* ≤ 0.05) compared to steers.

**Table 4.** Straight and branched-chain SFA composition of the LT muscle from steers and calves

	Steers		Calves		
%	Mean	SD	Mean	SD	*P*-value
∑SFA	45.8	2.5	43.9	3.1	0.039
14:0	2.43	0.40	4.03	0.63	≤0.001
15:0	0.546	0.063	0.680	0.076	≤0.001
16:0	25.1	2.1	24.0	1.6	0.118
17:0	1.09	0.12	1.07	0.12	0.522
18:0	15.9	1.4	13.3	0.8	≤0.001
19:0	0.118	0.020	0.125	0.018	0.293
20:0	0.125	0.015	0.114	0.014	0.029
∑BCFA	1.93	0.28	2.01	0.19	0.408
*i*-15:0	0.267	0.037	0.228	0.041	0.006
*i*-16:0	0.241	0.031	0.254	0.025	0.245
*i-*17:0	0.425	0.068	0.490	0.023	≤0.001
*i*-18:0	0.125	0.016	0.140	0.026	0.018
*a*-15:0	0.255	0.044	0.273	0.040	0.239
*a*-17:0	0.511	0.114	0.510	0.060	0.973

SD, standard deviation; SFA, saturated fatty acids; BCFA, branched-chain fatty acids; *i*, *iso*; *a*, *anteiso*.

**Table 5.** Monounsaturated and PUFA composition of the LT muscle from steers and calves

	Steers		Calves		
%	Mean	SD	Mean	SD	*P*-value
∑MUFA	39.1	2.1	34.3	1.8	≤0.001
∑cis-MUFA	36.4	2.1	32.1	1.8	≤0.001
9*c*-14:1	0.410	0.133	0.710	0.152	≤0.001
9*c*-15:1	0.143	0.047	0.207	0.056	≤0.001
7*c*-16:1	0.264	0.041	0.308	0.028	0.003
9*c*-16:1	2.46	0.50	2.19	0.23	0.018
9*c*-17:1	0.685	0.068	0.685	0.050	0.986
9*c*-18:1	29.8	1.8	25.3	1.6	≤0.001
11*c*-18:1	0.965	0.091	1.25	0.16	≤0.001
12*c*-18:1	0.0912	0.0139	0.110	0.025	0.003
13*c*-18:1	0.162	0.031	0.177	0.045	0.219
15*c*-18:1	0.135	0.021	0.0841	0.0108	≤0.001
11*c*-20:1	0.104	0.012	0.0979	0.0143	0.175
∑trans-MUFA	2.62	0.25	2.18	0.17	≤0.001
∑PUFA	7.65	2.29	11.9	2.9	≤0.001
∑n~-3	2.80	0.93	4.00	0.84	0.001
18:3n-3	1.13	0.32	1.42	0.25	0.012
20:4n-3	0.171	0.061	0.173	0.028	0.899
20:5n-3	0.595	0.268	1.03	0.28	≤0.001
22:5n-3	0.751	0.275	1.17	0.25	≤0.001
22:6n-3	0.115	0.050	0.166	0.065	0.009
∑n~-6	4.84	1.39	7.89	2.11	≤0.001
18:2n-6	3.31	0.89	5.36	1.51	≤0.001
20:3n-6	0.305	0.099	0.445	0.106	≤0.001
20:4n-6	1.07	0.41	1.88	0.47	≤0.001
n-6/n-3	1.75	0.158	1.96	0.191	≤0.001
P/S	0.170	0.0611	0.277	0.0932	≤0.001

SD, standard deviation; MUFA, monounsaturated fatty acids; *c*, *cis*; *t*, *trans*; PUFA, polyunsaturated fatty acids; P/S, PUFA/saturated fatty acids.

Total MUFA percentage and, specifically, the *cis*-MUFA and *trans*-MUFA were higher in the intramuscular fat of steers compared to calves (*P* ≤ 0.05; Table 5). Oleic and palmitoleic were the main *cis*-18:1 and *cis*-16:1 FA, respectively, and together with other minor FAs such as 15*c*-18:1, were higher in meat from steers than calves (*P* ≤ 0.05), whereas other minor *cis*-MUFA with different carbon chain lengths appeared to be higher in calf meat (Table 5). In terms of PUFA content, calf meat showed higher percentages compared to steers (*P* ≤ 0.05, Table 5), mainly due to the higher percentages of 18:3n-3, 20:5n-3, 22:5n-3, 22:6n-3, and almost all the reported n-6 FA (*P* ≤ 0.05) (Table 5). The n-6/n-3 and PUFA/SFA (P/S) ratios were also higher in the calf meat compared to steers (*P* ≤ 0.05; Table 5).

The contents of rumen biohydrogenation intermediates are shown in Figure 1. In general, all individual *trans*-18:1 isomers were higher in steers compared to calves (*P* ≤ 0.05), and vaccenic acid (VA; 11*t*-18:1) was the predominant isomer in both groups of animals followed by 13/14*t*-18:1 and 16*t*-18:1. In terms of CLA content, total CLA percentage was higher in calf compared to steer meat (*P* ≤ 0.05, Figure 1), mainly due to the higher content of rumenic acid (RA; 9*c*,11*t*-18:2) (*P* ≤ 0.05). Rumenic acid was the major CLA isomer of meat across treatments (Figure 1) and represented 60% of total CLA. The second main CLA (11*t*,13*c*-18:2) was higher in meat from steers than calves (*P* ≤ 0.05).

## DISCUSSION

As was expected, calves slaughtered at 10 mo of age, had lower carcass weights compared to steers. In general, concentrate finishing increases carcass weight when slaughtering animals at the same age. In this sense, Martin and Rogers (2004) found that grass-fed steers slaughtered at 18 to 20 mo of age provided lower carcass weights compared with more intensive finishing systems. Duckett et al. (2013) reported that, in steers finished to similar age, carcass from those finished on forage showed lower carcass weight, lower percentage of fat and higher percentage of bone than steers fed on concentrate.

The color of bovine subcutaneous carcass fat depends on the age and duration and type of feeding, among other factors (Dunne et al., 2009). In the present study, subcutaneous fat from steers was more yellow (higher b* value) than fat from calves. Those color differences could also be attributed in part to the age differences and the different animal diets. Beef quality measurements indicated that the color of meat from calves was lighter and less red compared to meat from steers. It is well documented that meat becomes darker and redder with increasing animal age (Faustman and Suman, 2017). On the other hand, concentrates have generally lower *b*-carotene contents than pasture, which leads to lower concentrations of this pigment in the fat of concentrate-fed animals as indicated by several authors (Knight et al., 1996; French et al., 2000; Varela et al., 2004; Dunne et al., 2009).

Shear force values were lower in meat from calves compared to steers mainly due to differences in animal age (calves were 8 to 10 mo younger than steers), although animal feeding could also explain some of the differences in shear force values. Brewer and Calkins (2003) stated that grain-fed beef has an advantage over grass-fed beef for sensory and instrumental tenderness, although some studies reported no effect of feeding regime (Muir et al., 1998; French et al., 2001). Shear force values from both treatments in this study are considered low and meat would be expected to be highly acceptable by most consumers, as meat tenderness is a key attribute defining consumer acceptability (Warren et al., 2008; Wood et al., 2008). Meat shear force values below 4.1 kg have been reported as satisfactory in eating quality by consumers (Huffman et al., 1996).

Grass-fed animals are frequently leaner than animals finished on grain (Van Elswyk and McNeill, 2014). In the present study meat from steers showed higher content of intramuscular fat compared to meat from calves. However, meat from both, steers and calves, was leaner in comparison with intramuscular fat content previously reported from steers finished on temperate pastures (2.21% to 2.42%; Morales et al., 2012, 2015), and from dairy bulls finished on concentrates (1.94%, Catrileo et al., 2014) in the South of Chile. The intramuscular fat content has been shown to be directly related to the extent of concentrate-finishing period (Wood et al., 2008). The accretion of intramuscular fat is associated with the increase in triacylglycerol, which would result in higher content of SFA and MUFA in finished beef (Rule et al., 1995). In the present study, calves showed higher percentages of PUFA and lower percentages of SFA and MUFA compared to steers due to the lower intramuscular fat level. On the other hand, phospholipid is an essential component of cell membranes and its amount remains fairly constant, or increases marginally, as the animal increases in fatness. In young lean animals, as is the case of calves in this study, the lower 9*c*-18:1 and higher 18:2 n-6 content of phospholipid has a major influence on total muscle FA composition (Wood et al., 2008). But as body fat increases, the proportion of phospholipid in total lipid decreases (from 30% at 14 mo to 12% at 24 mo, Warren et al., 2008) and this is accompanied by an increase in the proportion of 9*c*-18:1 and a decrease in the proportion of 18:2 n-6 in total lipids.

Rumenic acid (9*c*,11*t*-18:2) is produced as a result of the biohydrogenation process occurring in the rumen, where unsaturated FA (mainly 18:2n-6 and 18:3n-3) from the diet are first isomerized and partially saturated later (Christie, 1981). RA is also synthesized by endogenous conversion of VA by the enzyme Δ9-desaturase in the adipose tissue (Griinari and Bauman, 1999). Biohydrogenation decreases as the concentrate in the animal diet increases (Sauvant and Bas, 2001) and the RA concentration in the adipose tissue is higher when animals are fed on pasture than those fed on stored forages or grain (Realini et al., 2004; Nuernberg et al., 2005; Garcia et al., 2008; Leheska et al., 2008; De la Fuente et al., 2009). In the present study, calf meat showed a higher percentage of RA than meat from steers. Calves have been suckling on grazing cows and had access to forage while being supplemented with grain after weaning. It is well known that during the early stage of development, the reticular groove in calves is normally closed delivering suckled milk directly into the abomasum and bypassing the rumen (Hornick et al., 1997). Regarding the accumulation of other rumen biohydrogenation intermediates in meat, VA would have been the predominant *trans*-18:1 isomer in the milk because the cows were exclusively pasture-fed under Patagonian conditions with no access to concentrate. Consequently, considering the absence of a fully functioning rumen in young calves, the FA composition of cow’s milk would not be expected to be altered by rumen bacteria. Calves from the present study had free access to Patagonian pasture while suckling their mothers and supplemented with grain after weaning, which provided them with dietary 18:2n-6 and 18:3n-3. These PUFAs could be converted to either 11*t*- and/or 10*t*-18:1 in the developing rumen of the calves, depending on the amount of concentrate consumed. In several countries, recommendations for a healthy diet involve the reduction in the intake of *trans* fats (Aldai et al., 2013). For regulatory purposes, *trans*-FA is defined as *trans* monoenes plus other FA containing *trans* double bonds, except CLA (Aldai et al., 2011). Any food containing less than 0.2 g *trans*-FA and less than 0.5 g *trans*-FA per serving are considered free of *trans*-FA in the United States and Canada, respectively. The beef obtained in this study had a maximum content of 0.05 g *trans*-FA/100 g of meat that was well below the limit set either in Canada or in the United States. However, VA has been associated with health benefits (Kuhnt et al., 2016) and represents in the current study 42% and 47% of *trans*-MUFA in meat from steers and calves, respectively. In addition, it is considered that ruminant *trans* fats are generally not consumed in large amounts and have not been identified as representing a high risk of developing cardiovascular diseases (Gebauer et al., 2011; Wang et al., 2012). Consequently, they are exempt from *trans* labeling requirements because these FA sources are considered to be “natural” and therefore assumed to be “healthy,” especially VA and RA from animals raised on pasture (Aldai et al., 2013).

In general, the content of n-3 PUFAs in beef meat is low, even if animals are fed on pasture which is high in 18:3n-3 (De Smet et al., 2004; Scollan et al., 2006), because of extensive rumen biohydrogenation (Harfoot and Hazlewood, 1997). However, the results of this study showed that the content of total n-3 PUFAs, 18:3n-3, 20:5n-3, 22:5n-3, and 22:6n-3 in the meat was significantly higher from the calves compared to the meat from steers (Table 4). These results can be explained by the higher proportion of phospholipids in meat calves in comparison to steers.

Total fat content and the P/S and n-6/n-3 FA ratios are generally considered indicators of the nutritional value of fatty foods. As aforementioned, the fat content of the loin was very low in both types of animals and they could be considered as lean meat (<5% fat) according to USDA regulations. The P/S ratio of muscle fat was higher in the meat from calves than steers, although both values were within dietary recommendations (≥0.45, Department of Health, 1994). Demirel et al. (2006) indicated that it is difficult to increase the P/S ratio when ruminants are fed either grass hay or concentrate, whereas the n-6/n-3 ratio in meat is more influenced by the diet. Similar n-6/n-3 ratios have been reported by other authors in grass-fed beef (Schor et al., 2008; De la Fuente et al., 2009; Aldai et al., 2011; Morales et al., 2012, 2015).

There is limited nutritional information available characterizing the nutritional quality of Patagonian beef. Results from the present study provide quantitative information for human nutritionists to deliver more accurate consumer recommendations with values from local beef, rather than using data from international nutritional tables. In addition, beef production system in Patagonia is a pasture based system, which, in general, has nutrient limitations, affecting the system efficiency. The implementation of a more aggressive nutrition regime, will allow the rancher to finish the calve in a shorter period of time with a targeting supplementation, based on grain, to be able to obtain animals with the minimum subcutaneous fat required by the market, obtain the higher market value and improve animal welfare. The information resulting from the actual work on the nutritional quality of Patagonian beef from different production systems will be valuable for promoting beef consumption and local and international trade.

In summary, grain supplementation of calves in the Chilean Patagonia can reduce the finishing period on pasture by 8 to 10 mo, resulting in lighter carcasses but higher yield, lighter muscle color, lower yellowness in subcutaneous fat, and more tender meat compared to meat from pasture-finished steers. Loins from calves were leaner with higher proportions of healthy FA such as n-3 and CLA, being RA the main isomer, compared to meat from older and heavier pasture-fed steers. However, meat from steers finished on pasture was also tender and showed a FA profile within recommendations for a healthy diet. Thus, implementation of this alternative production system will mainly depend on the cost of grains compared with longer periods of pasture grazing and target carcass weights for defined markets.
